# American Medical Association, Section on Stomatology

**Published:** 1901-09

**Authors:** 


### Reports of Society Meetings.

### AMERICAN MEDICAL ASSOCIATION, SECTION ON STOMATOLOGY.

### DISCUSSION ON DEGENERACY OF THE PULP.

*Dr. R. R. Andrews,* Cambridge, Mass.—I will say in the first place that I was delighted with the paper by Dr. Latham,—with the care with which it was prepared and the suggestions which she made. I do not see how the subject could possibly have been treated any better or the photographs been made any more beautiful.

In regard to the paper of our secretary concerning this matter of preparing tissue, I have tried all these various complicated methods, and with more or less success, but I finally came to the conclusion that if I got my tissue as nearly alive as possible, and used the simplest possible means of preparing it, I got in every case the better results. I have worked mostly on the heads of calves, because it seemed to me they showed the tissue so well. I have taken the calf at birth, taken out the teeth, and transferred them to a low per cent. of chromic acid solution, one-half of one per cent., and if necessary I would use some stronger acids, hydrochloric, for instance, a very small per cent. I cannot tell just exactly the combination which I used. I placed the tissue in the solution and tested it from day to day, and as soon as I found the area of the tooth sufficiently softened to use the microtome I would cut sections until my microtome reached hard tissue. Many sections which show well with low powers are almost useless when used for high-power work. The tissue seems to run together, and there is no definite picture and no differentiation of the tissue. Sections for high-power work have got to be more carefully prepared, and it takes a long time. I have always been in favor of working as near life as possible soon after preparation was taken from the live body, and in the simplest possible way.

I have been very much pleased with the character of the papers that have been read, not only to-day, but yesterday also.

*Dr. G. V. I. Brown,* Milwaukee, Wis.—The symposium interested me a great deal, although I have somewhat of a feeling of disappointment that we could not have gone on now from where we left off and put in at least a day in pursuing this subject a little farther. I would have liked to see it developed along the line of pathological changes that occur in the pulp, so that under the microscope we could arrive at some definite etiological idea. I have had a great many interesting cases the past year, but in a large measure the benefit of them has been lost, because in our laboratories in Milwaukee we do not seem to have any distinguishing way of arriving definitely at the pathological changes that take place in the pulp-tissue. I have in mind one case in which there was a spasmodic affection of the muscles of the face, accompanied by a recurrent pain which occurred at intervals of one and one-half to two minutes, so severe that the life of the patient was almost unendurable and the hyperæsthesia at the posterior portion of the mouth and pharynx made it almost impossible to take nourishment. The difficulty was finally located in the tooth-pulp and cured, and although that pulp was carefully preserved and an attempt made to treat it so it would show the characteristics of that condition, we were unable to arrive at anything which we felt we could give out as scientifically correct. That is the reason why I think the effect of this symposium will be far-reaching, and we will hear more of it, not only in this section, but in other societies, and I hope what has been begun to-day will be carried farther before this question is dropped.

*Dr. R. R. Andrews,* Cambridge, Mass.—If Dr. Talbot, in the large number of teeth he examined, had made a careful study of them, it might have been of great benefit in this direction, and he would have found that the pulp even at full maturity of the tooth is of great service. It is a mistake to suppose that the pulp has lost its function when it is fully formed. I know it is the belief of many that when teeth are fully formed the pulp might as well be killed, but those who have studied this subject most will tell you that a healthy dental pulp has a function to perform through life.

*Dr. Eugene S. Talbot,* Chicago.—I hardly think the chairman and Dr. Brown have quite grasped the situation in regard to these papers. The general subject of this symposium is "Preliminary Work." The teeth selected by myself were taken at all stages by men who make a business of extracting teeth. Age was hence not taken into consideration in a general way. The object of the paper was to give my own results only. Dr. Latham, Dr. Anderson, and myself have undertaken a large amount of work, which is all preliminary, in a systematic way, taking up the general conditions of the teeth in a healthy state and from embryologic and nosologic points of view. There is one point in dental literature to which attention was paid, giving credit to whom credit is due. One subject of these papers is to summarize the literature. One-half of it has been given. The idea is to compile all literature upon the pulp before generalizing. That has been my method throughout life. All my writings show that I feel obligated to give due credit to those that have done work before. After that I take up the line of work, and in all the points where the work corresponds with that previously done to give due credit. If it differ, I show wherein difference occurs. That is the only way in which to make scientific research.

*Dr. Vida A. Latham,* Rogers Park, Ill.—I would like to say that I am in the centre of a large city, but I have had extreme difficulty in getting hold of material. Our library boasts of a medical department, but it is almost devoid of dental literature except a few current journals. The Northwestern Dental School has a library, but it is rather difficult of access, and then the papers are either in Swedish or Hungarian, and I am ignorant of those languages. I have tried to be pretty careful in that regard, and I have found papers that were under men's names that were little familiar, and when I made a careful search I have found they were not their papers at all. So I have tried to be careful on that point. It is rather difficult to follow the literature. I have used the *Cosmos* index, and it is a very excellent one, and I have used Dr. Taft's index, which is also a very good one, and in that way I have not missed any good articles in the journals, and I would be glad to hear of any good papers that have been published.

There is another point in pulp methods that Dr. Anderson has brought out. I have tried to use simple methods. I believe the only successful methods that are being used are those which take in the jaw itself. It is almost useless to take out the tooth, because you break the pulp and the continuity is broken. It is difficult to get a jaw or teeth from the human unless you are attached to a hospital. There is a prejudice in America against the post mortem; it is almost impossible to procure teeth. A point that strikes me very forcibly is, Why should we use the term "degenerate" in the embryo? Take the new works on embryology, take the American works, take any of the standard works, and you will find nothing of degenerate pulp; the name does not occur. When I think of the importance of it, when the literature is silent on it, how shall we treat secondary dentine? Why should we take out the pulp? How can we treat the pulp when we do not know what it is? It is impossible. Out of ten pulps, nine are pathological. I have tried to follow them down with hydrochloric acid. The men who do dental histological work take the old methods. Dental histologic methods must be studied with dental tissues. What have we got to experiment with? Dr. Anderson is very fond of chlorine, but after a few years the specimens will disappear. My specimens that I mounted ten years ago I can find no trace of, and under those conditions I feel a little doubtful about using chlorine. A man may write to you asking if you have certain slides, and therefore you must keep the specimens. I have written to Dr. Sudduth for specimens, and he has replied that he did not keep his slides, as they were so easy to get. Personally I do not agree with him. I have put in six months on one slide, and then had difficulty to secure it. This is mostly preliminary work, and now we expect to go to work with calves, with the embryo from the stock-yards, and find out where the pulp comes from. Some say it forms the odontoblasts, and some say it does not. If the secondary dentine exists, there must be secondary and not primary nerves.

*Dr. G. V. I. Brown,* Milwaukee, Wis.—I am glad to hear what Dr. Latham has said, and am also glad to hear the statement about the preparation of specimens. So far as I have tried the work it has been unsatisfactory. A large proportion of the methods are comes from. Some say it forms the odontoblasts, and some say it does not. If the secondary dentine exists, there must be secondary effect in demonstration; and then you must have a perfect specimen.

*Dr. R. R. Andrews,* Cambridge, Mass.—Just one word in regard to Morgenstern's demonstration. I think he used the Golgi method. He found that the dentinal fibrils within the dentinal canals were wound about by delicate fibres that had every appearance of being delicate nerve-fibrils coming from the pulp. I worked with this method for more than six months with entirely negative results. It shows that it is a difficult problem to solve.

